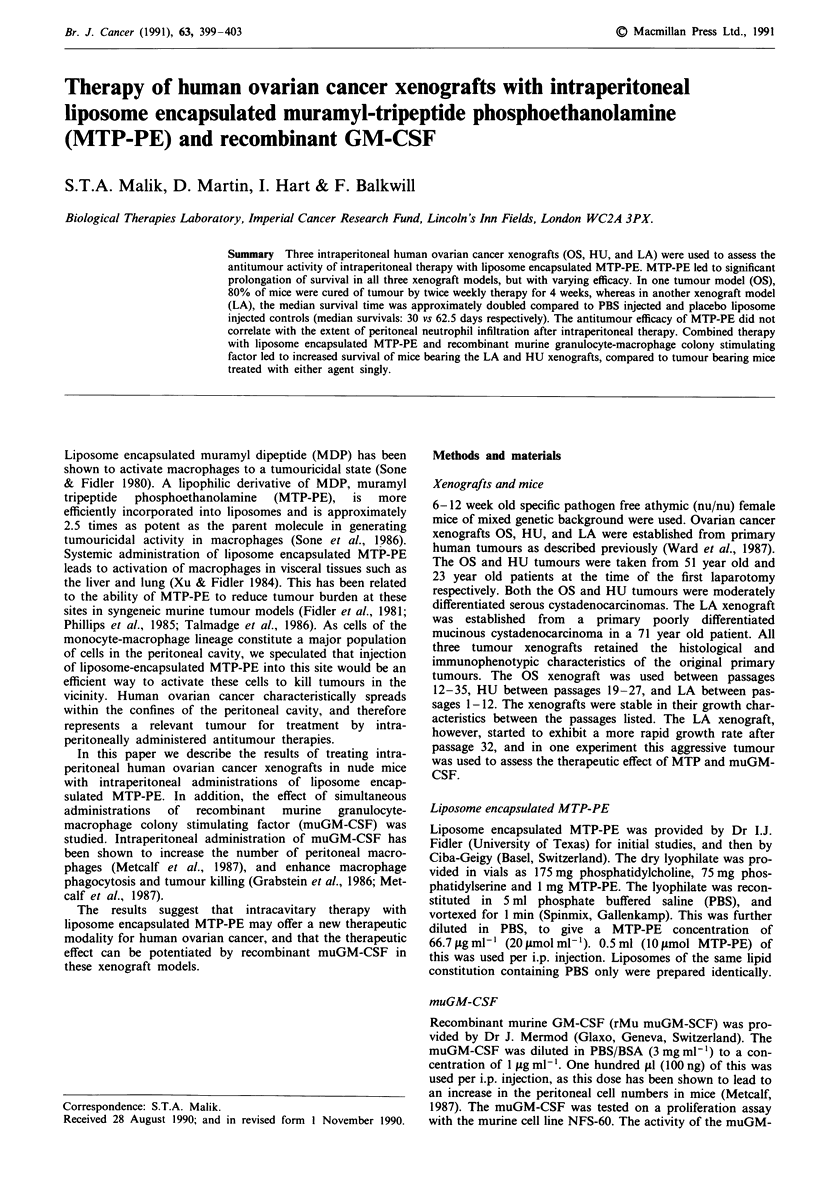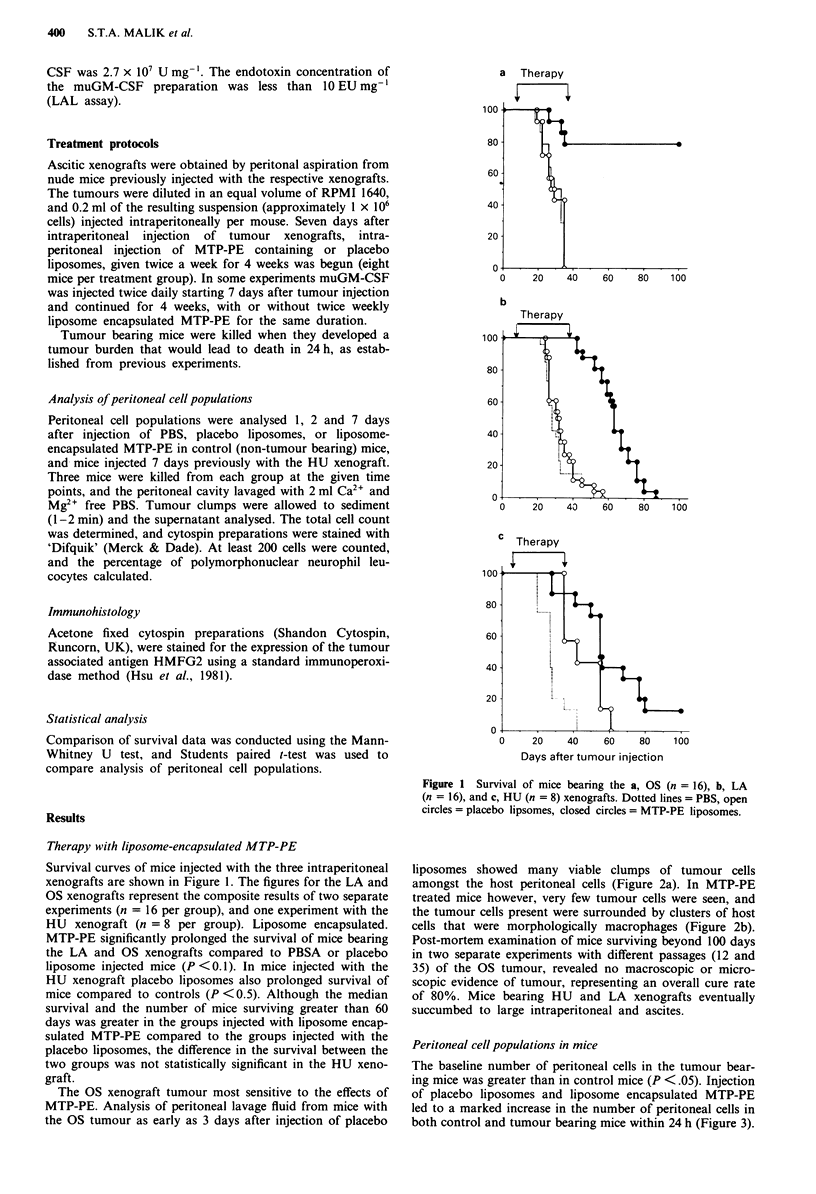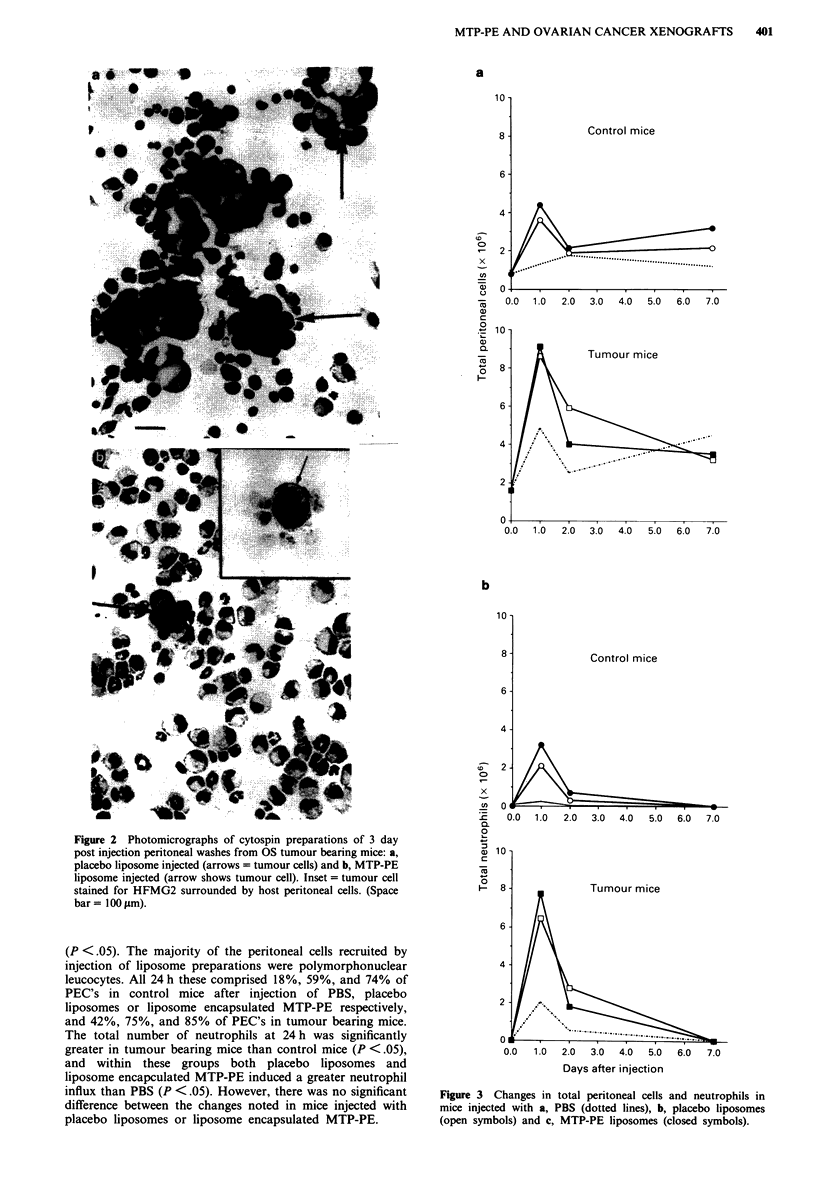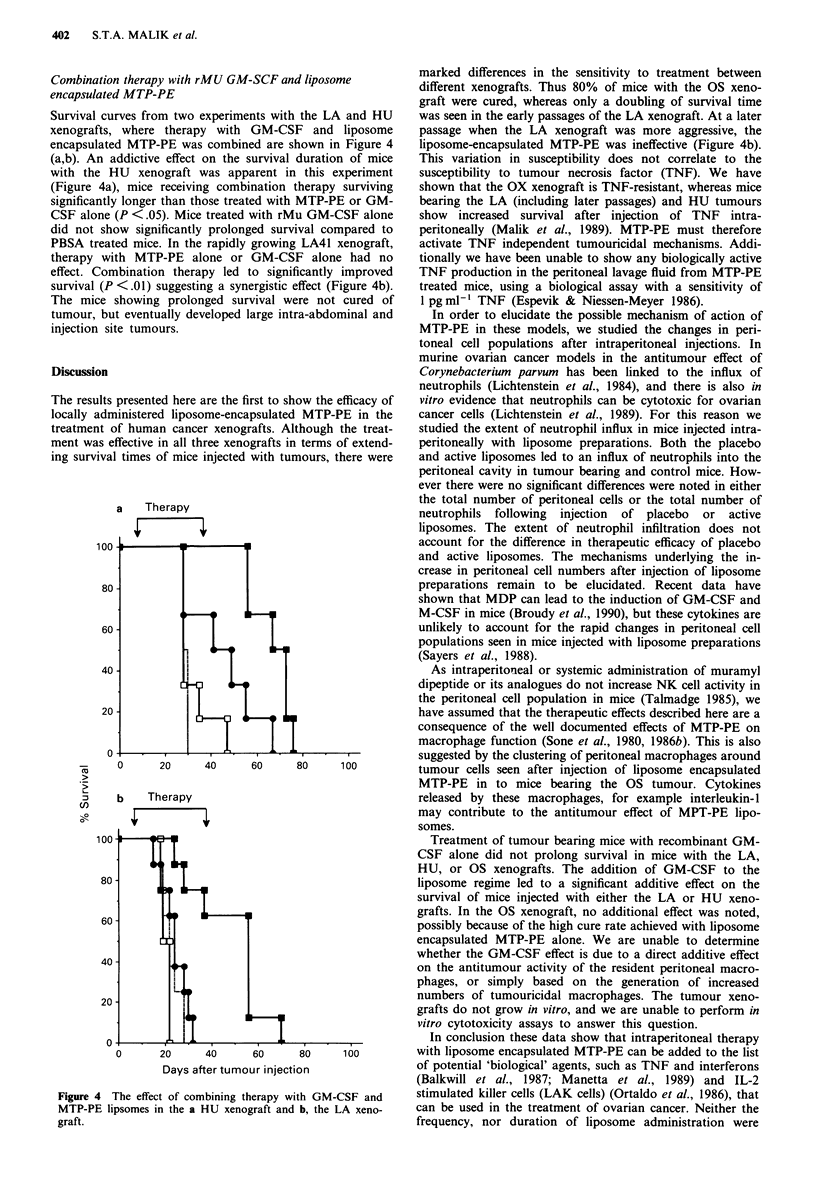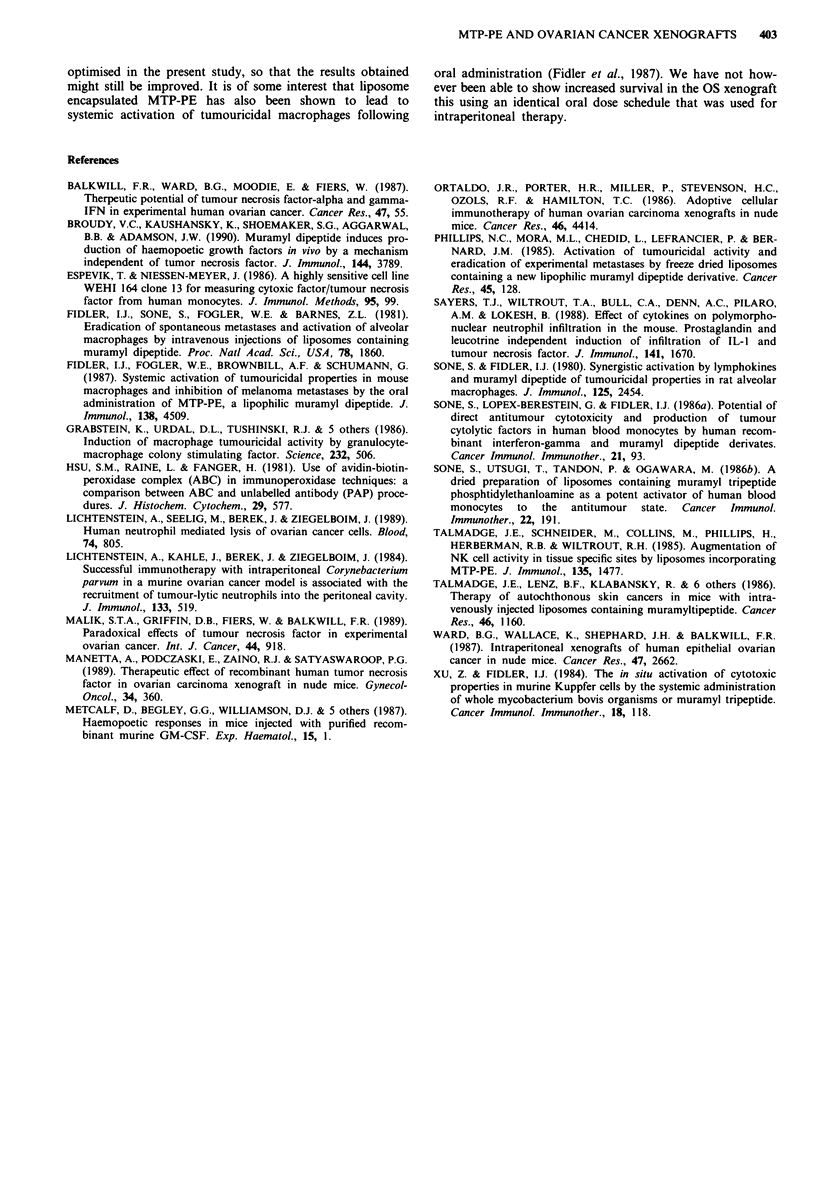# Therapy of human ovarian cancer xenografts with intraperitoneal liposome encapsulated muramyl-tripeptide phosphoethanolamine (MTP-PE) and recombinant GM-CSF.

**DOI:** 10.1038/bjc.1991.92

**Published:** 1991-03

**Authors:** S. T. Malik, D. Martin, I. Hart, F. Balkwill

**Affiliations:** Biological Therapies Laboratory, Imperial Cancer Research Fund, Lincoln's Inn Fields, London.

## Abstract

**Images:**


Br. J. Cancer (1991), 63, 399-403
© Macmillan Press Ltd., 1991

### Therapy of human ovarian cancer xenografts with intraperitoneal liposome encapsulated muramyl-tripeptide phosphoethanolamine (MTP-PE) and recombinant GM-CSF

S.T.A. Malik, D. Martin, I. Hart & F. Balkwill

*Biological Therapies Laboratory, Imperial Cancer Research Fund, Lincoln's Inn Fields, London WC2A 3PX.*

**Summary** Three intraperitoneal human ovarian cancer xenografts (OS, HU, and LA) were used to assess the antitumour activity of intraperitoneal therapy with liposome encapsulated MTP-PE. MTP-PE led to significant prolongation of survival in all three xenograft models, but with varying efficacy. In one tumour model (OS), 80% of mice were cured of tumour by twice weekly therapy for 4 weeks, whereas in another xenograft model (LA), the median survival time was approximately doubled compared to PBS injected and placebo liposome injected controls (median survivals: 30 *vs* 62.5 days respectively). The antitumour efficacy of MTP-PE did not correlate with the extent of peritoneal neutrophil infiltration after intraperitoneal therapy. Combined therapy with liposome encapsulated MTP-PE and recombinant murine granulocyte-macrophage colony stimulating factor led to increased survival of mice bearing the LA and HU xenografts, compared to tumour bearing mice treated with either agent singly.

Liposome encapsulated muramyl dipeptide (MDP) has been shown to activate macrophages to a tumouricidal state (Sone & Fidler 1980). A lipophilic derivative of MDP, muramyl tripeptide phosphoethanolamine (MTP-PE), is more efficiently incorporated into liposomes and is approximately 2.5 times as potent as the parent molecule in generating tumouricidal activity in macrophages (Sone *et al.*, 1986). Systemic administration of liposome encapsulated MTP-PE leads to activation of macrophages in visceral tissues such as the liver and lung (Xu & Fidler 1984). This has been related to the ability of MTP-PE to reduce tumour burden at these sites in syngeneic murine tumour models (Fidler *et al.*, 1981; Phillips *et al.*, 1985; Talmadge *et al.*, 1986). As cells of the monocyte-macrophage lineage constitute a major population of cells in the peritoneal cavity, we speculated that injection of liposome-encapsulated MTP-PE into this site would be an efficient way to activate these cells to kill tumours in the vicinity. Human ovarian cancer characteristically spreads within the confines of the peritoneal cavity, and therefore represents a relevant tumour for treatment by intraperitoneally administered antitumour therapies.

In this paper we describe the results of treating intraperitoneal human ovarian cancer xenografts in nude mice with intraperitoneal administrations of liposome encapsulated MTP-PE. In addition, the effect of simultaneous administrations of recombinant murine granulocyte-macrophage colony stimulating factor (muGM-CSF) was studied. Intraperitoneal administration of muGM-CSF has been shown to increase the number of peritoneal macrophages (Metcalf *et al.*, 1987), and enhance macrophage phagocytosis and tumour killing (Grabstein *et al.*, 1986; Metcalf *et al.*, 1987).

The results suggest that intracavitary therapy with liposome encapsulated MTP-PE may offer a new therapeutic modality for human ovarian cancer, and that the therapeutic effect can be potentiated by recombinant muGM-CSF in these xenograft models.

Correspondence: S.T.A. Malik.
Received 28 August 1990; and in revised form 1 November 1990.

### Methods and materials

### *Xenografts and mice*

6-12 week old specific pathogen free athymic (nu/nu) female mice of mixed genetic background were used. Ovarian cancer xenografts OS, HU, and LA were established from primary human tumours as described previously (Ward *et al.*, 1987). The OS and HU tumours were taken from 51 year old and 23 year old patients at the time of the first laparotomy respectively. Both the OS and HU tumours were moderately differentiated serous cystadenocarcinomas. The LA xenograft was established from a primary poorly differentiated mucinous cystadenocarcinoma in a 71 year old patient. All three tumour xenografts retained the histological and immunophenotypic characteristics of the original primary tumours. The OS xenograft was used between passages 12-35, HU between passages 19-27, and LA between passages 1-12. The xenografts were stable in their growth characteristics between the passages listed. The LA xenograft, however, started to exhibit a more rapid growth rate after passage 32, and in one experiment this aggressive tumour was used to assess the therapeutic effect of MTP and muGM-CSF.

### *Liposome encapsulated MTP-PE*

Liposome encapsulated MTP-PE was provided by Dr I.J. Fidler (University of Texas) for initial studies, and then by Ciba-Geigy (Basel, Switzerland). The dry lyophilate was provided in vials as 175 mg phosphatidylcholine, 75 mg phosphatidylserine and 1 mg MTP-PE. The lyophilate was reconstituted in 5 ml phosphate buffered saline (PBS), and vortexed for 1 min (Spinmix, Gallenkamp). This was further diluted in PBS, to give a MTP-PE concentration of 66.7 $\mu g\,ml^{-1}$ (20 $\mu mol\,ml^{-1}$). 0.5 ml (10 $\mu$mol MTP-PE) of this was used per i.p. injection. Liposomes of the same lipid constitution containing PBS only were prepared identically.

### *muGM-CSF*

Recombinant murine GM-CSF (rMu muGM-SCF) was provided by Dr J. Mermod (Glaxo, Geneva, Switzerland). The muGM-CSF was diluted in PBS/BSA (3 $mg\,ml^{-1}$) to a concentration of 1 $\mu g\,ml^{-1}$. One hundred $\mu$l (100 ng) of this was used per i.p. injection, as this dose has been shown to lead to an increase in the peritoneal cell numbers in mice (Metcalf, 1987). The muGM-CSF was tested on a proliferation assay with the murine cell line NFS-60. The activity of the muGM-

CSF was $2.7 \times 10^7$ U $mg^{-1}$. The endotoxin concentration of the muGM-CSF preparation was less than 10 EU $mg^{-1}$ (LAL assay).

### Treatment protocols

Ascitic xenografts were obtained by peritonal aspiration from nude mice previously injected with the respective xenografts. The tumours were diluted in an equal volume of RPMI 1640, and 0.2 ml of the resulting suspension (approximately $1 \times 10^6$ cells) injected intraperitoneally per mouse. Seven days after intraperitoneal injection of tumour xenografts, intraperitoneal injection of MTP-PE containing or placebo liposomes, given twice a week for 4 weeks was begun (eight mice per treatment group). In some experiments muGM-CSF was injected twice daily starting 7 days after tumour injection and continued for 4 weeks, with or without twice weekly liposome encapsulated MTP-PE for the same duration.

Tumour bearing mice were killed when they developed a tumour burden that would lead to death in 24 h, as established from previous experiments.

### *Analysis of peritoneal cell populations*

Peritoneal cell populations were analysed 1, 2 and 7 days after injection of PBS, placebo liposomes, or liposome-encapsulated MTP-PE in control (non-tumour bearing) mice, and mice injected 7 days previously with the HU xenograft. Three mice were killed from each group at the given time points, and the peritoneal cavity lavaged with 2 ml $Ca^{2+}$ and $Mg^{2+}$ free PBS. Tumour clumps were allowed to sediment (1–2 min) and the supernatant analysed. The total cell count was determined, and cytospin preparations were stained with 'Difquik' (Merck & Dade). At least 200 cells were counted, and the percentage of polymorphonuclear neurophil leucocytes calculated.

### *Immunohistology*

Acetone fixed cytospin preparations (Shandon Cytospin, Runcorn, UK), were stained for the expression of the tumour associated antigen HMFG2 using a standard immunoperoxidase method (Hsu *et al.*, 1981).

### *Statistical analysis*

Comparison of survival data was conducted using the Mann-Whitney U test, and Students paired *t*-test was used to compare analysis of peritoneal cell populations.

**Figure 1** Survival of mice bearing the **a**, OS ($n$ = 16), **b**, LA ($n$ = 16), and **c**, HU ($n$ = 8) xenografts. Dotted lines = PBS, open circles = placebo liposomes, closed circles = MTP-PE liposomes.

### Results

### *Therapy with liposome-encapsulated MTP-PE*

Survival curves of mice injected with the three intraperitoneal xenografts are shown in Figure 1. The figures for the LA and OS xenografts represent the composite results of two separate experiments ($n$ = 16 per group), and one experiment with the HU xenograft ($n$ = 8 per group). Liposome encapsulated. MTP-PE significantly prolonged the survival of mice bearing the LA and OS xenografts compared to PBSA or placebo liposome injected mice ($P < 0.1$). In mice injected with the HU xenograft placebo liposomes also prolonged survival of mice compared to controls ($P < 0.5$). Although the median survival and the number of mice surviving greater than 60 days was greater in the groups injected with liposome encapsulated MTP-PE compared to the groups injected with the placebo liposomes, the difference in the survival between the two groups was not statistically significant in the HU xenograft.

The OS xenograft tumour most sensitive to the effects of MTP-PE. Analysis of peritoneal lavage fluid from mice with the OS tumour as early as 3 days after injection of placebo liposomes showed many viable clumps of tumour cells amongst the host peritoneal cells (Figure 2a). In MTP-PE treated mice however, very few tumour cells were seen, and the tumour cells present were surrounded by clusters of host cells that were morphologically macrophages (Figure 2b). Post-mortem examination of mice surviving beyond 100 days in two separate experiments with different passages (12 and 35) of the OS tumour, revealed no macroscopic or microscopic evidence of tumour, representing an overall cure rate of 80%. Mice bearing HU and LA xenografts eventually succumbed to large intraperitoneal and ascites.

### *Peritoneal cell populations in mice*

The baseline number of peritoneal cells in the tumour bearing mice was greater than in control mice ($P < .05$). Injection of placebo liposomes and liposome encapsulated MTP-PE led to a marked increase in the number of peritoneal cells in both control and tumour bearing mice within 24 h (Figure 3).

**Figure 2** Photomicrographs of cytospin preparations of 3 day post injection peritoneal washes from OS tumour bearing mice: **a**, placebo liposome injected (arrows = tumour cells) and **b**, MTP-PE liposome injected (arrow shows tumour cell). Inset = tumour cell stained for HFMG2 surrounded by host peritoneal cells. (Space bar = 100 μm).

($P < .05$). The majority of the peritoneal cells recruited by injection of liposome preparations were polymorphonuclear leucocytes. All 24 h these comprised 18%, 59%, and 74% of PEC's in control mice after injection of PBS, placebo liposomes or liposome encapsulated MTP-PE respectively, and 42%, 75%, and 85% of PEC's in tumour bearing mice. The total number of neutrophils at 24 h was significantly greater in tumour bearing mice than control mice ($P < .05$), and within these groups both placebo liposomes and liposome encapculated MTP-PE induced a greater neutrophil influx than PBS ($P < .05$). However, there was no significant difference between the changes noted in mice injected with placebo liposomes or liposome encapsulated MTP-PE.

**Figure 3** Changes in total peritoneal cells and neutrophils in mice injected with **a**, PBS (dotted lines), **b**, placebo liposomes (open symbols) and **c**, MTP-PE liposomes (closed symbols).

*Combination therapy with rMU GM-SCF and liposome encapsulated MTP-PE*

Survival curves from two experiments with the LA and HU xenografts, where therapy with GM-CSF and liposome encapsulated MTP-PE was combined are shown in Figure 4 (a,b). An addictive effect on the survival duration of mice with the HU xenograft was apparent in this experiment (Figure 4a), mice receiving combination therapy surviving significantly longer than those treated with MTP-PE or GM-CSF alone ($P < .05$). Mice treated with rMu GM-CSF alone did not show significantly prolonged survival compared to PBSA treated mice. In the rapidly growing LA41 xenograft, therapy with MTP-PE alone or GM-CSF alone had no effect. Combination therapy led to significantly improved survival ($P < .01$) suggesting a synergistic effect (Figure 4b). The mice showing prolonged survival were not cured of tumour, but eventually developed large intra-abdominal and injection site tumours.

### Discussion

The results presented here are the first to show the efficacy of locally administered liposome-encapsulated MTP-PE in the treatment of human cancer xenografts. Although the treatment was effective in all three xenografts in terms of extending survival times of mice injected with tumours, there were marked differences in the sensitivity to treatment between different xenografts. Thus 80% of mice with the OS xenograft were cured, whereas only a doubling of survival time was seen in the early passages of the LA xenograft. At a later passage when the LA xenograft was more aggressive, the liposome-encapsulated MTP-PE was ineffective (Figure 4b). This variation in susceptibility does not correlate to the susceptibility to tumour necrosis factor (TNF). We have shown that the OX xenograft is TNF-resistant, whereas mice bearing the LA (including later passages) and HU tumours show increased survival after injection of TNF intraperitoneally (Malik *et al.*, 1989). MTP-PE must therefore activate TNF independent tumouricidal mechanisms. Additionally we have been unable to show any biologically active TNF production in the peritoneal lavage fluid from MTP-PE treated mice, using a biological assay with a sensitivity of 1 pg $ml^{-1}$ TNF (Espevik & Niessen-Meyer 1986).

In order to elucidate the possible mechanism of action of MTP-PE in these models, we studied the changes in peritoneal cell populations after intraperitoneal injections. In murine ovarian cancer models in the antitumour effect of *Corynebacterium parvum* has been linked to the influx of neutrophils (Lichtenstein *et al.*, 1984), and there is also *in vitro* evidence that neutrophils can be cytotoxic for ovarian cancer cells (Lichtenstein *et al.*, 1989). For this reason we studied the extent of neutrophil influx in mice injected intraperitoneally with liposome preparations. Both the placebo and active liposomes led to an influx of neutrophils into the peritoneal cavity in tumour bearing and control mice. However there were no significant differences were noted in either the total number of peritoneal cells or the total number of neutrophils following injection of placebo or active liposomes. The extent of neutrophil infiltration does not account for the difference in therapeutic efficacy of placebo and active liposomes. The mechanisms underlying the increase in peritoneal cell numbers after injection of liposome preparations remain to be elucidated. Recent data have shown that MDP can lead to the induction of GM-CSF and M-CSF in mice (Broudy *et al.*, 1990), but these cytokines are unlikely to account for the rapid changes in peritoneal cell populations seen in mice injected with liposome preparations (Sayers *et al.*, 1988).

As intraperitoneal or systemic administration of muramyl dipeptide or its analogues do not increase NK cell activity in the peritoneal cell population in mice (Talmadge 1985), we have assumed that the therapeutic effects described here are a consequence of the well documented effects of MTP-PE on macrophage function (Sone *et al.*, 1980, 1986*b*). This is also suggested by the clustering of peritoneal macrophages around tumour cells seen after injection of liposome encapsulated MTP-PE in to mice bearing the OS tumour. Cytokines released by these macrophages, for example interleukin-1 may contribute to the antitumour effect of MPT-PE liposomes.

Treatment of tumour bearing mice with recombinant GM-CSF alone did not prolong survival in mice with the LA, HU, or OS xenografts. The addition of GM-CSF to the liposome regime led to a significant additive effect on the survival of mice injected with either the LA or HU xenografts. In the OS xenograft, no additional effect was noted, possibly because of the high cure rate achieved with liposome encapsulated MTP-PE alone. We are unable to determine whether the GM-CSF effect is due to a direct additive effect on the antitumour activity of the resident peritoneal macrophages, or simply based on the generation of increased numbers of tumouricidal macrophages. The tumour xenografts do not grow *in vitro*, and we are unable to perform *in vitro* cytotoxicity assays to answer this question.

In conclusion these data show that intraperitoneal therapy with liposome encapsulated MTP-PE can be added to the list of potential 'biological' agents, such as TNF and interferons (Balkwill *et al.*, 1987; Manetta *et al.*, 1989) and IL-2 stimulated killer cells (LAK cells) (Ortaldo *et al.*, 1986), that can be used in the treatment of ovarian cancer. Neither the frequency, nor duration of liposome administration were

**Figure 4** The effect of combining therapy with GM-CSF and MTP-PE lipsomes in the **a** HU xenograft and **b**, the LA xenograft.

optimised in the present study, so that the results obtained might still be improved. It is of some interest that liposome encapsulated MTP-PE has also been shown to lead to systemic activation of tumouricidal macrophages following oral administration (Fidler *et al.*, 1987). We have not however been able to show increased survival in the OS xenograft this using an identical oral dose schedule that was used for intraperitoneal therapy.

### References

BALKWILL, F.R., WARD, B.G., MOODIE, E. & FIERS, W. (1987). Therpeutic potential of tumour necrosis factor-alpha and gamma-IFN in experimental human ovarian cancer. *Cancer Res.*, **47**, 55.

BROUDY, V.C., KAUSHANSKY, K., SHOEMAKER, S.G., AGGARWAL, B.B. & ADAMSON, J.W. (1990). Muramyl dipeptide induces production of haemopoetic growth factors *in vivo* by a mechanism independent of tumor necrosis factor. *J. Immunol.*, **144**, 3789.

ESPEVIK, T. & NIESSEN-MEYER, J. (1986). A highly sensitive cell line WEHI 164 clone 13 for measuring cytoxic factor/tumour necrosis factor from human monocytes. *J. Immunol. Methods*, **95**, 99.

FIDLER, I.J., SONE, S., FOGLER, W.E. & BARNES, Z.L. (1981). Eradication of spontaneous metastases and activation of alveolar macrophages by intravenous injections of liposomes containing muramyl dipeptide. *Proc. Natl Acad. Sci., USA*, **78**, 1860.

FIDLER, I.J., FOGLER, W.E., BROWNBILL, A.F. & SCHUMANN, G. (1987). Systemic activation of tumouricidal properties in mouse macrophages and inhibition of melanoma metastases by the oral administration of MTP-PE, a lipophilic muramyl dipeptide. *J. Immunol.*, **138**, 4509.

GRABSTEIN, K., URDAL, D.L., TUSHINSKI, R.J. & 5 others (1986). Induction of macrophage tumouricidal activity by granulocyte-macrophage colony stimulating factor. *Science*, **232**, 506.

HSU, S.M., RAINE, L. & FANGER, H. (1981). Use of avidin-biotin-peroxidase complex (ABC) in immunoperoxidase techniques: a comparison between ABC and unlabelled antibody (PAP) procedures. *J. Histochem. Cytochem.*, **29**, 577.

LICHTENSTEIN, A., SEELIG, M., BEREK, J. & ZIEGELBOIM, J. (1989). Human neutrophil mediated lysis of ovarian cancer cells. *Blood*, **74**, 805.

LICHTENSTEIN, A., KAHLE, J., BEREK, J. & ZIEGELBOIM, J. (1984). Successful immunotherapy with intraperitoneal *Corynebacterium parvum* in a murine ovarian cancer model is associated with the recruitment of tumour-lytic neutrophils into the peritoneal cavity. *J. Immunol.*, **133**, 519.

MALIK, S.T.A., GRIFFIN, D.B., FIERS, W. & BALKWILL, F.R. (1989). Paradoxical effects of tumour necrosis factor in experimental ovarian cancer. *Int. J. Cancer*, **44**, 918.

MANETTA, A., PODCZASKI, E., ZAINO, R.J. & SATYASWAROOP, P.G. (1989). Therapeutic effect of recombinant human tumor necrosis factor in ovarian carcinoma xenograft in nude mice. *Gynecol-Oncol.*, **34**, 360.

METCALF, D., BEGLEY, G.G., WILLIAMSON, D.J. & 5 others (1987). Haemopoetic responses in mice injected with purified recombinant murine GM-CSF. *Exp. Haematol.*, **15**, 1.

ORTALDO, J.R., PORTER, H.R., MILLER, P., STEVENSON, H.C., OZOLS, R.F. & HAMILTON, T.C. (1986). Adoptive cellular immunotherapy of human ovarian carcinoma xenografts in nude mice. *Cancer Res.*, **46**, 4414.

PHILLIPS, N.C., MORA, M.L., CHEDID, L., LEFRANCIER, P. & BERNARD, J.M. (1985). Activation of tumouricidal activity and eradication of experimental metastases by freeze dried liposomes containing a new lipophilic muramyl dipeptide derivative. *Cancer Res.*, **45**, 128.

SAYERS, T.J., WILTROUT, T.A., BULL, C.A., DENN, A.C., PILARO, A.M. & LOKESH, B. (1988). Effect of cytokines on polymorphonuclear neutrophil infiltration in the mouse. Prostaglandin and leucotrine independent induction of infiltration of IL-1 and tumour necrosis factor. *J. Immunol.*, **141**, 1670.

SONE, S. & FIDLER, I.J. (1980). Synergistic activation by lymphokines and muramyl dipeptide of tumouricidal properties in rat alveolar macrophages. *J. Immunol.*, **125**, 2454.

SONE, S., LOPEX-BERESTEIN, G. & FIDLER, I.J. (1986*a*). Potential of direct antitumour cytotoxicity and production of tumour cytolytic factors in human blood monocytes by human recombinant interferon-gamma and muramyl dipeptide derivates. *Cancer Immunol. Immunother.*, **21**, 93.

SONE, S., UTSUGI, T., TANDON, P. & OGAWARA, M. (1986*b*). A dried preparation of liposomes containing muramyl tripeptide phosphtidylethanloamine as a potent activator of human blood monocytes to the antitumour state. *Cancer Immunol. Immunother.*, **22**, 191.

TALMADGE, J.E., SCHNEIDER, M., COLLINS, M., PHILLIPS, H., HERBERMAN, R.B. & WILTROUT, R.H. (1985). Augmentation of NK cell activity in tissue specific sites by liposomes incorporating MTP-PE. *J. Immunol.*, **135**, 1477.

TALMADGE, J.E., LENZ, B.F., KLABANSKY, R. & 6 others (1986). Therapy of autochthonous skin cancers in mice with intravenously injected liposomes containing muramyltipeptide. *Cancer Res.*, **46**, 1160.

WARD, B.G., WALLACE, K., SHEPHARD, J.H. & BALKWILL, F.R. (1987). Intraperitoneal xenografts of human epithelial ovarian cancer in nude mice. *Cancer Res.*, **47**, 2662.

XU, Z. & FIDLER, I.J. (1984). The *in situ* activation of cytotoxic properties in murine Kuppfer cells by the systemic administration of whole mycobacterium bovis organisms or muramyl tripeptide. *Cancer Immunol. Immunother.*, **18**, 118.